# Polymer Nanocomposites: Preparation, Characterisation and Applications

**DOI:** 10.3390/nano12111900

**Published:** 2022-06-01

**Authors:** Kinga Pielichowska

**Affiliations:** Department of Biomaterials and Composites, Faculty of Materials Science and Ceramics, AGH University of Science and Technology, Al. Mickiewicza 30, 30-059 Kraków, Poland; kingapie@agh.edu.pl

Polymer nanocomposites are an interesting and rapidly growing class of novel materials with enhanced properties, and these enhancements can be observed even at low nanofiller loading. Polymer nanocomposites are considered—due to their properties—as promising materials in a wide variety of applications, including automotive, aerospace, mechanics, electronics [1], catalysis [2], food packing [3], medicine [4] and environment protection [5,6].

The structure, properties, and their relationships in polymer nanocomposites strongly depend not only on the type of polymer matrix, its average molar mass/dispersity and the architecture of polymer chains, but also on the type, chemical composition, shape, size and content of nanoparticles and their compatibility with the polymer matrix [7]. Proper functionalization of nanomaterials can substantially improve polymer–filler compatibility thus preventing or limiting undesired agglomeration effects and lead to enhanced properties. Depending on these factors, nanoparticles can strongly influence mechanical, rheological, and optical properties; flame retardancy; and the thermal and electrical conductivity of polymer matrices. It is also well-known that nanoparticles can change the thermal stability and degradation mechanism (including kinetics) of a polymer matrix [8,9]. They may influence the mechanism of polymer matrix crystallization and facilitate the formation of new crystalline structures [10].

This Special Issue of Nanomaterials covers the most recent advances in the area of polymer nanocomposites, including their preparation, compatibilization and processing, along with properties and methods of their characterization. It also reports on nanoeffects such as entanglements, confinement and other phenomena connected to the incorporation of nanosize particles to polymer matrix, and their influence on the behaviour of macromolecules. Papers on applications of polymer nanocomposites in different sectors, ranging from mechanical engineering, automotive, and buildings to electronics and biomedicine, have been published in this Special Issue.

Hence, Han et al. [11] investigated the effects of graphene on the microstructure, mechanical, electrical, and thermal properties of the graphene/polyethylene (PE) nanocomposites by using positron annihilation lifetime spectroscopy (PALS). FTIR results revealed the formation of CH-π interactions between PE and graphene during processing. Authors postulated that the CH-π interactions can serve as a sacrificial bond to dissipate stress. The highest values of tensile strength (11.35 MPa) were found for composites modified with 0.25 wt% graphene. With increasing graphene content from 0 to 2.0 wt%, the relative free volume fraction decreased from 31.63% to 28.38%, making the movement of the polymer chain more difficult and leading to an increase of the melting temperature of PE from 109 °C to 112 °C. Moreover, as the low free volume fraction can prevent heat transfer and the diffusion of pyrolysis products, the highest thermal stability was obtained for the composites possessing the lowest free volume fraction (2.0 wt% graphene/PE). The results obtained gave deeper insight into the relationships between structure and performance for polymer composites with nanosized fillers.

Lu et al. [12] studied natural antimicrobial nanocomposite fibres obtained from an alginate and oregano essential oil (OEO). It has been demonstrated that by using the electrospinning method sodium alginate-based nanofibers with incorporated OEO can be obtained. The production method of alginate nanofibers has been described: in the first step, crosslinking of alginate was performed, while in the second step deionized water soaking was applied. Microscopic investigations enabled to determine sodium alginate/OEO nanofiber diameters which was in the range of 38 and 105 nm. The preliminary antimicrobial activity assessment revealed that sodium alginate/OEO nanofibres inhibited the growth of Gram-positive and Gram-negative bacterial wounds and foodborne pathogens. Overall, this study demonstrated that OEO could be incorporated within alginate nanofibres while maintaining physical and antimicrobial properties. Future applications may include antimicrobial medical textiles in biomedical and food packing sectors.

Method of quantitative visualization of Young’s modulus of soft materials by atomic force microscopy (AFM) was presented by Kim et al. [13]. Authors measured Young’s modulus values of various polymeric materials at each pixel within the entire scanning area and implemented the quantitative visualization of the mechanical properties. The presented method in the PinPointTM nanomechanical mode allows direct visualization of Young’s modulus, surface topography and statistical analysis of measured values at each pixel.

Abualnaja et al. [14] impregnated (acrylonitrile-co-styrene) P(AN-co-St) composite with adsorbents, such as sulfonated and multiwall carbon nanotubes (MWCNTs), to increase the adsorption ability of the nanocomposite upon the removal of methyl orange (MO) dye. Changes in MO adsorption on the three nanocomposites were examined in an aqueous solution. The obtained results show that the efficiency of P(AN-co-St)/MWCNT removal increased under the conditions of an acidic pH; the obtained composites can be used as efficient materials for the adsorption of MO dye from aqueous solutions.

Graphene oxide (GO) and nanoclay-based filler in chlorobutyl-natural rubber (CIIR/NR) blends were used by Paduvilan et al. [15] for advanced gas barrier applications. Rheological studies revealed that the curing time decreases at higher filler load. The increase in modulus was observed after incorporation of GO and nanoclay to the CIIR/NR matrix along with. The systems with carbon black and nanoclay exhibited a gradual increase in modulus with an increase of filler content. At higher fillers load composites gas impermeability was reduced due to agglomeration of nanofillers.

Jeong et al. [16] described a fabrication method of elastomeric conductive skin-like composite. E-skin substrates were obtained using polydimethylsiloxane (PDMS) and photosensitive polyimide (PSPI). A flexible strain sensor for e-skin was obtained by the formation of laser-induced graphene (LIG) on the skin-like substrates. The skin-like strain sensor was characterized by good ultra-wide sensing range, large sensitivity, short response time, and recovery time. The authors concluded that the proposed sensor has potential for application in wearable health monitoring devices and human–machine interface systems.

Generalized approach for mechanical properties evaluation of polymer nanocomposites with spherical fillers based on a three-phase series-parallel model was presented by Martinez-Garcia et al. [17]. In the proposed model, authors considered percolation and the glassy interphase between filler and matrix. The proposed model has been validated with experimental data obtained for various polymer nanocomposites.

Visco et al. [18] studied polyurethane foams modified with carbon nanofibers (CN) to enhance the selectivity and mechanical durability of the PU foams in relation to oils. They revealed that incorporation of CN leads to an increase in the hydrophobic behaviour and a good oleophilic character of the composite sponges. The best properties were found for composites containing 1 wt% of nanofiller.

The effect of incorporation of poly(ε-caprolactone) (PCL) functionalized nanohydroxyapatite (HAp) into the polyoxymethylene copolymer (POM) was investigated by Pielichowska et al. [19]. It has been showed that the incorporation of functionalized HAp into the POM matrix has a limited effect on the POM phase transitions and degree of crystallinity, but significantly increases the thermal stability of the POM. Crystallization kinetic studies showed that in small amounts functionalized HAp can act as a nucleating agent for the POM crystallization process. Furthermore, functionalized HAp improved the in vitro bioactivity of the polyoxymethylene composites that is crucial for orthopaedic applications.

In a review paper by Rennhofer and Zanghellini [20] dispersion state as well as damage of carbon nanotubes (CNT) and CN by sonication was described. Sonication is one of the most widely used methods for dispersion of nanoadditives in liquid systems, such as solvents, prepolymers, or resins. However, sonication can potentially damage the liquid system (e.g., through polymer chain scission) or CNT by scission, surface damage, or both. This review gives a current state of the art on dispersion of carbon-based nanoadditives by sonication and can help readers in future studies to access key information related to specific sonication strategies.

I hope this Special Issue will contribute to a better understanding of polymer-based nanocomposite structure and properties, thus opening further application perspectives of this promising class of materials.
